# Neurologists' approaches and challenges in managing early-stage Alzheimer's disease: A survey of clinical practices

**DOI:** 10.6026/9732063002001954

**Published:** 2024-12-31

**Authors:** Devupriya Mohanan Sujatha, Siraj Muhammed Shafy, Ashish Abraham Francis, Vishal Rajkumar, Khushii Durvasula Jagdeesh, Shreya Krishna, Sanjit Pradeep, Nikitha Gudapati

**Affiliations:** 1 Department of General Medicine, Joondalup Health Campus, Perth, Western Australia; 2 Department of Neurosurgery, VPS Lakeshore Hospital, Kochi, Kerala, India; 3 Institute of Internal Medicine, Madras Medical College, Chennai, India; 4 Institute of Internal Medicine, Madras Medical College, Chennai, India; 5 Department of Medicine, Sri Ramachandra Institute of Higher Education and Research, Chennai, India; 6 Department of Internal Medicine, Sri Ramachandra Institute of Higher Education and Research, Chennai, India; 7 Department of Internal Medicine, Rajiv Gandhi Government General Hospital, Chennai, Tamil Nadu, India

**Keywords:** Alzheimer's disease, early-stage management, neurologists, clinical practices, survey-based study

## Abstract

Alzheimer's disease (AD), a progressive neurodegenerative disorder, requires early intervention to delay cognitive decline and enhance quality of life. This national survey of 100 neurologists explored their clinical practices in managing early-stage AD, including diagnostic approaches, treatment selection, patient counseling and perceived barriers. While neurologists acknowledged the importance of early intervention, challenges such as limited resources, time constraints and patient non-cooperation hindered optimal care. Variability in diagnostic and counseling practices often stemmed from disparities in resources and training. These findings highlight the need for revised guidelines, enhanced training and improved resources to support neurologists in providing consistent and effective early-stage AD care.

## Background:

Alzheimer's disease is a progressive neurodegenerative condition, characterized by cognitive deterioration and loss of memory and operative capacity. It is in this sense that it represents one of the most important public health challenges being faced at the moment; it goes hand in hand with population aging and the expected growth in its incidence. It has declared that Alzheimer's disease is the most common cause, according to the World Health Organization; over 55 million people have dementia. Early diagnosis and treatment is the cornerstone since interventions at preliminary stages of AD have shown potential to decelerate disease progression and improve quality of life among patients [1, 2]. Indeed, in the medical world, neurologists would be quite different because it would involve making a diagnosis and handling early-stage AD. The time of minor changes is the hardest to grasp both in terms of its duration and onset. Neurologists use neuroimaging tests, cognitive testing and biomarkers to determine a diagnosis and progress. It consists of non-pharmacologic interventions like strategies of early intervention adopted to improve cognitive function, relieve symptoms and thus help patients and their caregivers [3, 4]. Pharmacologically, they are cholinesterase inhibitors and NMDA receptor antagonists. Cognitive therapy, exercise and nutritional changes are related to the non-pharmacological approach [5, 6]. Despite clear guidelines recommending holistic management of AD, neurologists are severely restricted by several factors that limit them from providing uniform and best possible care. These include the fact that not enough time is available for the consultation in the absence of adequate caregiver support resources and non-cooperation of the patients. The variability concerning access to advanced diagnostic tests such as biomarkers, which is more often recommended but not universally available, is also incredibly significant [7, 8]. Due to the constant new developments in AD research and changes in diagnostic and treatment guidelines, practice by neurologists is always in a state of needing education and advancement. The other key stage of management integration is patient and caregiver counselling with AD management. A better counselling approach will be able to address what patients are worried about, how and what they ought to do about their treatment and educate their caregivers on how to manage their symptoms and prepare them for the future. Neurologists may not be professionally trained in behavioural interventions or are constrained by systemic regulations that dictate how much time is spent on counselling in a clinic visit [9, 10]. For such complexities, the understanding of the current practice, challenges and educational needs of the neurologists offering early-stage AD care becomes quite important. Therefore, it is of interest to provide detailed insight into the approaches neurologists have toward early-stage AD, considering practice-pattern variations, diagnostic tools, preferred treatments and perceived challenges. From the research findings, areas and challenges that need to be improved will be identified, along with recommendations that may help them provide quality and standard care to those patients who are at the initial stage of Alzheimer's disease in diverse health setups [11].

## Methodology:

A cross-sectional design involving a survey-based assessment of neurologists' practices, approaches and difficulties in dealing with early-stage Alzheimer's disease (AD) was the research method. The objective of the survey was to obtain thorough insights into clinical strategies employed at the diagnostic stage by neurologists in treating early AD, counselling patients and managing early-stage AD, as well as common barriers toward optimal care. A structured questionnaire was formulated based on a literature review and consultations with experts to reflect relevance and clarity. The questionnaire incorporated questions on demographic and practice characteristics, clinical diagnostic approaches, treatment preferences and perceived challenges in managing early-stage AD. Questions on demographics captured data regarding years of experience, the setting for practice and regions. Subsequent sections touched on particular clinical practices, for instance application of cognition tests, pharmacological and non-pharmacological interventions follow up with the patient and usual challenges experienced, such as the poor adherence by the patient or the unavailability of support resources. The target population was 100 neurological practitioners working in diverse practice environments across the country. The requirement for inclusion criteria was to have a minimum period of one year of clinical exposure in neurology. The questionnaire was available through an online, password-protected site and anonymous to ensure that neurologists were able to access and complete it as conveniently as possible. Data collection lasted for three months. Reminder emails were sent every two weeks to maximize response rates. Response was entirely voluntary and all participants gave informed consent. Data analysis was therefore conducted in SPSS version 25.0, from which descriptive statistics such as means, standard deviations and frequency distributions of the demographic variables and the responses to the survey were generated. Inferential statistics, including t-tests and ANOVA, were used to analyse differences among approaches and challenges based on years of experience and practice setting. Institutional review board had given ethical clearance to conduct the study and confidentiality was ensured throughout the study.

## Questionnaire:

The questionnaire consisted of both closed-ended and open-ended questions. Questions dealing with diagnostic criteria, treatment preferences and perceived barriers were close-ended, while the open-ended ones gav

Table 1 below shows the Summary of the Questionnaire Structure: The questionnaire examines neurologists' clinical practices and challenges in managing early-stage Alzheimer's disease, focusing on diagnostic approaches, treatment strategies and patient management. It includes questions on the use of diagnostic tools, criteria for early detection, preferred therapeutic options and barriers to implementing evidence-based practices. The survey also explores neurologists' perspectives on patient education, caregiver involvement and access to resources, aimed at identifying gaps and opportunities for improving early-stage Alzheimer's care.

## Results:

Table 2 outlines the demographic profile, including age, years of practice and practice setting, providing a snapshot of the neurologist population in this study. Table 3 summarizes the diagnostic criteria used by neurologists, including reliance on biomarkers, imaging and cognitive testing. Table 4 shows neurologists' preferred medications for managing early-stage AD, highlighting cholinesterase inhibitors as the most common choice. Table 5 presents the types of non-pharmacologic interventions neurologists recommend, including cognitive therapy and lifestyle changes. Table 6 indicates the frequency with which neurologists counsel patients and caregivers, underscoring the emphasis on education.

Table 7 details the primary barriers neurologists face in managing early-stage AD, including limited time and patient compliance issues. Table 8 shows neurologists' perceptions of the importance of early intervention in AD, with a strong consensus on its value. Table 9 highlights neurologists' interest in further training for managing AD, particularly in areas of diagnostic advancements and patient counselling. Table 10 presents neurologists' access to resources, showing a need for improved support materials and specialist collaboration. Table 11 summarizes open-ended responses, with neurologists highlighting specific challenges in providing comprehensive care for early-stage AD.

## Discussion:

It mentions various strategies in the treatment of early-stage Alzheimer's disease, efforts and difficulties neurologists face. Imaging and neuropsychological tests were the most used to make a diagnosis, though few reported accesses to advanced diagnostic tools such as biomarkers and genetic testing was limited. This article is according to current literature as multimodal diagnostics are especially important for the detection of early AD, but access could depend on different practice settings [12]. Pharmacologic treatments, especially cholinesterase inhibitors, are universally applied, but non-pharmacologic measures like cognitive therapy and exercise contribute essential components to care plans. Still, there are a range of obstacles including lack of time, uncooperative patients and resource-poor families which often prevent neurologists from fully pursuing these measures [13]. Many respondents wished for more institutional sources, such as the availability of specialized facilities or allied health professionals; to augment the scope of care provided [14]. Of particular interest is the desire for additional education or training in AD management among neurologists [15]. This suggests that the educational programs the neurologists have received thus far may not be adequate to prepare individuals handling this extraordinarily complex condition [16]. Additional education and training could inform neurologists of the latest diagnostic and therapeutic approaches, with a direct impact on patient care [17]. The open-ended questions allow for richer responses, where neurologists point out the importance of caregiver support groups and multidisciplinary teamwork in order to cope with the wide range of problems presented by AD [18, 19]. Involvement of social workers, psychologists and other professionals working in AD can lead to further enriched support for both patients and their caregivers [20]. VR training could improve the condition and cognitive skills of AD patients [21].

The patient should always be given information about follow-up and post-diagnostic care. Lastly, advice on brain-healthy behaviour and attention to modifiable risk factors can help to empower the patient to do something themselves to influence the disease course [22]. This study highlights the urgent necessity of comprehensive support for neurologists in the care of early-stage AD, including updated guidelines, easily accessible tools and resources and interdisciplinary collaboration [20]. The above needs may lead to more standardized and effective management practices, thereby improving the quality of life of patients with AD and their families. The survey data are self-reported and thus may suffer from response bias. Although the sample was nationally representative, further research could explore regional resource availability and practice patterns for a more complete understanding.

## Conclusion:

This study accepts that neurologists faced such challenges while dealing with early Alzheimer's disease patients, which were resource inadequacy, compliance by the patient and required more training. Neurologists show a keen interest in having further training and institutional support towards building a commitment to enhance early AD care. Stronger institutional support with better coordination and consultation towards a multi-disciplinary team may help increase access towards all the facilities of diagnosis and therapy, hence strengthen their capabilities in effective patient-centric care for AD.

## Figures and Tables

**Table 1:** Summary of the questionnaire

**Section**	**Focus Area**	**Details Included**
Demographics	Background information	Age, years in practice, practice setting
Diagnostic Approaches	Criteria for diagnosing AD	Use of biomarkers, imaging, neuropsychological testing
Treatment Modalities	Preferred pharmacologic and non-pharmacologic interventions	Medications, cognitive therapy, lifestyle modifications
Counselling Practices	Patient and caregiver guidance	Frequency of counselling sessions, topics covered
Barriers to Effective Management	Challenges in delivering care	Time constraints, lack of resources, patient compliance issues
Education and Training Needs	Interest in further training on AD management	Willingness to participate in training on AD diagnosis and treatment
Open-Ended Responses	Additional insights from neurologists	Please share any specific challenges or insights related to managing early-stage AD

**Table 2:** Demographic characteristics of neurologist

**Variable**	**Percentage (%)**
Age 25-35	22
Age 36-45	40
Age 46-55	28
Age 56+	10
< 5 Years' Experience	15
5-15 Years' Experience	45
> 15 Years' Experience	40
Academic Setting	35
Private Practice	45
Community Health Centre	20

**Table 3:** Diagnostic approaches for early-stage Alzheimer's disease

**Diagnostic Tool**	**Percentage (%)**
Biomarker Testing	55
Imaging (MRI/CT)	70
Neuropsychological Testing	65
Genetic Testing	15

**Table 4:** Preferred pharmacologic interventions

**Medication Type**	**Percentage (%)**
Cholinesterase Inhibitors	75
NMDA Receptor Antagonists	40
Antidepressants	25
Anxiolytics	10

**Table 5:** Non-pharmacologic intervention

**Intervention**	**Percentage (%)**
Cognitive Therapy	65
Physical Activity	50
Dietary Modifications	40
Social Engagement Activities	30

**Table 6:** Frequency of patient and caregiver counselling

**Counselling Frequency**	**Percentage (%)**
Every Visit	30
Monthly	35
Quarterly	25
Annually	10

**Table 7:** Barriers to effective AD management

**Barrier**	**Percentage (%)**
Limited Time	60
Patient Compliance Issues	45
Limited Access to Resources	40
Caregiver Support Challenges	30

**Table 8:** Neurologists' attitudes toward early-stage AD management

**Attitude**	**Percentage (%)**
Very Important	72
Important	25
Neutral	3

**Table 9:** Interest in additional training

**Interest in Training**	**Percentage (%)**
Very Interested	60
Somewhat Interested	30
Not Interested	10

**Table 10:** Availability of resources for AD management

**Resource Availability**	**Percentage (%)**
Adequate	35
Limited	50
Not Available	15

**Table 11:** Common themes from open-ended responses

**Theme**	**Percentage of Responses (%)**
Need for More Resources	45
Demand for Specialized Training	30
Challenges with Patient and Caregiver Compliance	25
